# 115 Overtreatment of Clostridioides difficile Colonization for Patients Residing in Davidson County, Tennessee from 2018–2022

**DOI:** 10.1017/ash.2026.10530

**Published:** 2026-06-23

**Authors:** Rajasekhar Jagarlamudi, Melissa Jackson, Jeremie Sawadogo, Michael Kent

**Affiliations:** 1 JPS Health System; 2 JPS Health Network

## Abstract

**Background:** Current antimicrobial and epidemiology literature has shown that most antibiotics are prescribed in the outpatient setting. Therefore, antimicrobial stewardship programs play a significant role in the reduction of inappropriate antibiotic utilization. JPS Health Network antimicrobial stewardship team presented provider education to the network’s community health clinics, aimed at reducing inappropriate antibiotic utilization in the outpatient setting. Providers receiving education in these community clinics included MD/DOs, medical residents, and advanced practice providers. Along with education, antibiotic prescribing scorecards were sent to individual clinic directors, allowing for direct comparison of individual clinics’ antibiotic prescribing patterns for sinusitis. The present quality improvement initiative aims at assessing the impact of these interventions on antibiotic prescribing patterns for sinusitis at JPS Health Network. Primary objective: Determine the rate of patients who received antibiotics for sinusitis at JPS Health Network’s community health clinics setting before and after provider education and implementation of sinusitis scorecards. **Methods:** Retrospective observational data was collected from the Electronic Health Record database (Epic) as part of the quality improvement initiative to assess antibiotics prescribing rate in patient diagnosed with sinusitis (ICD10-CM code included any variation of “J01” (acute sinusitis) or “J32” (chronic sinusitis) both before the interventions were implemented (September 1, 2024 through November 30, 2024) and after the interventions were implemented (January 1, 2025 through March 31, 2025). **Results:** A total of 1,807 patient encounters with sinusitis were recorded during the specified time frame. Of these, 151 patient encounters were excluded due to concurrent infections. A total of 603 antibiotics were prescribed in the preintervention group out of 788 patient encounters (77%) and 640 antibiotics were prescribed in the post-intervention group out of 868 patient encounters (74%) (p antibiotics prescribed for sinusitis. **Conclusions:** The antimicrobial stewardship interventions at the JPS Health Network’s community health clinics did result in a decrease in the overall prescribing of antibiotics for sinusitis. This finding reveals the significant role that antimicrobial stewardship program can play in outpatient settings to reduce the antibiotic utilization. Further research into outpatient antimicrobial stewardship interventions is warranted.

